# Housing and health outcomes: evidence on child morbidities from six Sub-Saharan African countries

**DOI:** 10.1186/s12887-023-03992-5

**Published:** 2023-05-05

**Authors:** Kanyiva Muindi, Samuel Iddi, Hellen Gitau, Blessing Mberu

**Affiliations:** 1 Population Dynamics and Urbanization, African Population and Health Research Center, Nairobi, Kenya; 2 Department of Statistics and Actuarial Science, University of Ghana, Legon, Accra Ghana; 3 Demography and Population Studies, University of Witwatersrand, Johannesburg, South Africa

**Keywords:** Africa, Diarrhoea, Acute respiratory illness, Morbidity, Housing

## Abstract

**Background:**

The connection between healthy housing status and health is well established. The quality of housing plays a significant role in infectious and non-communicable as well as vector-borne diseases. The global burden of disease attributable to housing is considerable with millions of deaths arising from diarrheal and respiratory diseases annually. In sub-Saharan Africa (SSA), the quality of housing remains poor although improvements have been documented. There is a general dearth of comparative analysis across several countries in the sub-region. We assess in this study, the association between healthy housing and child morbidity across six countries in SSA.

**Methods:**

We use the Demographic and Health Survey (DHS) data for six countries where the most recent survey collected health outcome data on child diarrhoea, acute respiratory illness, and fever. The total sample size of 91,096 is used in the analysis (representing 15, 044 for Burkina Faso, 11, 732 for Cameroon, 5, 884 for Ghana, 20, 964 for Kenya, 33, 924 for Nigeria, and 3,548 for South Africa). The key exposure variable is healthy housing status. We control for various factors associated with the three childhood health outcomes. These include quality housing status, residency (rural/urban), age of the head of the household, mother’s education, mother’s BMI status, marital status, mother’s age, and religious status. Others include the child’s gender, age, whether the child is from multiple or single births, and breastfeeding status. Inferential analysis using survey-weighted logistic regression is employed.

**Results:**

Our findings indicate that housing is an important determinant of the three outcomes investigated. Compared to unhealthier housing, healthy housing status was found to be associated with reduced odds of diarrhoea in Cameroon [Healthiest: aOR = 0.48, 95% CI, (0.32,0.71), healthier: aOR = 0.50, 95% CI,(0.35,0.70), Healthy: aOR = 0.60, 95% CI, (0.44,0.83), Unhealthy: aOR = 0.60, 95% CI, (0.44,0.81)], Kenya [Healthiest: aOR = 0.68, 95% CI, (0.52,0.87), Healtheir: aOR = 0.79, 95% CI, (0.63,0.98), Healthy: aOR = 0.76, 95% CI, (0.62,0.91)], South Africa[Healthy: aOR = 0.41, 95% CI, (0.18, 0.97)], and Nigeria [Healthiest: aOR = 0.48, 95% CI,(0.37,0.62), Healthier: aOR = 0.61, 95% CI,(0.50,0.74), Healthy: aOR = 0.71, 95%CI, (0.59,0.86), Unhealthy: aOR = 0.78, 95% CI, (0.67,0.91)], and reduced odds of Acute Respiratory Infection in Cameroon [Healthy: aOR = 0.72, 95% CI,(0.54,0.96)], Kenya [Healthiest: aOR = 0.66, 95% CI, (0.54,0.81), Healthier: aOR = 0.81, 95% CI, (0.69,0.95)], and Nigeria [Healthiest: aOR = 0.69, 95% CI, (0.56,0.85), Healthier: aOR = 0.72, 95% CI, (0.60,0.87), Healthy: aOR = 0.78, 95% CI, (0.66,0.92), Unhealthy: aOR = 0.80, 95% CI, (0.69,0.93)] while it was associated with increased odds in Burkina Faso [Healthiest: aOR = 2.45, 95% CI, (1.39,4.34), Healthy: aOR = 1.55, 95% CI, (1.09,2.20)] and South Africa [Healthy: aOR = 2.36 95% CI, (1.31, 4.25)]. In addition, healthy housing was significantly associated with reduced odds of fever among children in all countries except South Africa [Healthiest: aOR = 2.09, 95% CI, (1.02, 4.29)] where children living in the healthiest homes had more than double the odds of having fever. In addition, household-level factors such as the age of the household head, and place of residence were associated with the outcomes. Child-level factors such as breastfeeding status, age, and sex, and maternal-level factors such as education, age, marital status, body mass index (BMI), and religion were also associated with the outcomes.

**Conclusions:**

The dissimilarity of findings across similar covariates and the multiple relations between healthy housing and under 5 morbidity patterns show unequivocally the heterogeneity that exists across African countries and the need to account for different contexts in efforts to seek an understanding of the role of healthy housing in child morbidity and general health outcomes.

**Supplementary Information:**

The online version contains supplementary material available at 10.1186/s12887-023-03992-5.

## Background

Housing is a social determinant of health and remains an important factor in the overall well-being of occupants. The WHO guidelines on housing and health describe attributes of healthy housing that are critical to the well-being of occupants. These include the physical housing structure, the local community that enables social interactions that foster health, the immediate housing environment, and ‘the extent to which it offers services, green spaces, active transport and protection from pollution, waste, and protection from effects of disasters [1]. A recent analysis of healthy housing in Sub-Saharan Africa (SSA) indicates that rural areas have higher proportions of healthy housing compared to urban areas, with wealthier households having higher proportions of healthy housing compared to poor households [2]. While there have been improvements in the access to quality housing in SSA over the years, a significant proportion of the urban and rural population remain in poor housing [3], compromising the health of millions.

Quality of housing has been associated with various health outcomes such as infectious diseases [4, 5], vector-borne diseases [6, 7], and non-communicable diseases including mental health [1]. The burden of disease attributable to housing is considerable, with 1.6 million deaths in 2016 arising from diarrheal disease (accounting for 1.9% of the global burden of disease), driven by poor access to Water, Sanitation, and Hygiene (WASH) services. Most of this burden is in Asia and SSA [8]. On the other hand, respiratory diseases account for more than 10% of disability-adjusted life years (DALYs), with an estimated 4 million deaths annually arising from chronic respiratory diseases. Further, 9 million children below the age of 5 years die each year from respiratory illnesses [9]. A study on Tuberculosis (TB) epidemics indicates the importance of housing quality in terms of space (as a measure of crowding) and indoor pollution as a proximate risk factor, being in the causal pathway between poverty and TB risk [4].

Improving various aspects of housing quality has been suggested as one way to ensure the health and wellbeing of occupants [1, 10, 11]. Indeed, studies demonstrate that improving housing quality such as installation of screens is protective against malaria [6, 7], diarrheal diseases with improvement of floors [12], reduced risks of waterborne diseases in Ahmedabad [13], and reduced child mortality in Karachi both from improvement in access to sanitation [14]. While a recent study in SSA did not observe any specific association between housing conditions and acute respiratory infections (ARI), it reported that children living in housing with improved drinking water and sanitation, sufficient living area, and durable construction had a reduced likelihood of four major causes of death: malaria (12–18% reduction), diarrhoea (8% reduction), growth failure (stunting:17% reduction, wasting: 10% reduction, underweight: 15% reduction), and anaemia (11–13% reduction) [15]. Further, studies evaluating improved housing intervention in Latin America found a reduction in respiratory, diarrheal diseases as well as skin infections in children. Housing improvements were additionally associated with improved mental health, as well as social relationships [16].

Beyond health, housing quality has impacts on education and subsequent economic outcomes, especially for children [11]. A study across 20 SSA countries found children from improved housing 15% more likely to be developmentally on track in the cognitive domain [17]. Similarly, there are improved perceptions of safety, arising from improvements to the environment [16].

Building on the relevance of these associations for policy and action around the social determinants of health in the SSA region, and against the backdrop of the dearth of comparative data across several African countries, we undertake this study to assess the association between healthy housing and morbidity outcomes of diarrhoea, acute respiratory illness (ARI), and fever among children below five years of age across six African countries. The significance of this study is to contribute to the attainment of SDG 3 on health and well-being through evidence generation on the role of housing in childhood illness. Further, it will draw attention to housing attributes, especially access to water, sanitation, and hygiene (SDG 6) as well as household energy (SDG 7) as determinants of health that can be addressed to help countries achieve SDG 3.

## Conceptual framework

This paper is guided by housing and healthy child development frameworks by Dunn [18], which has six key housing attributes namely: (1) Biological, chemical, and physical hazards; (2) Physical design; (3) Psychological attributes; (4) Social attributes; (5) Financial attributes; (6) Locational attributes.

**Fig. 1:**
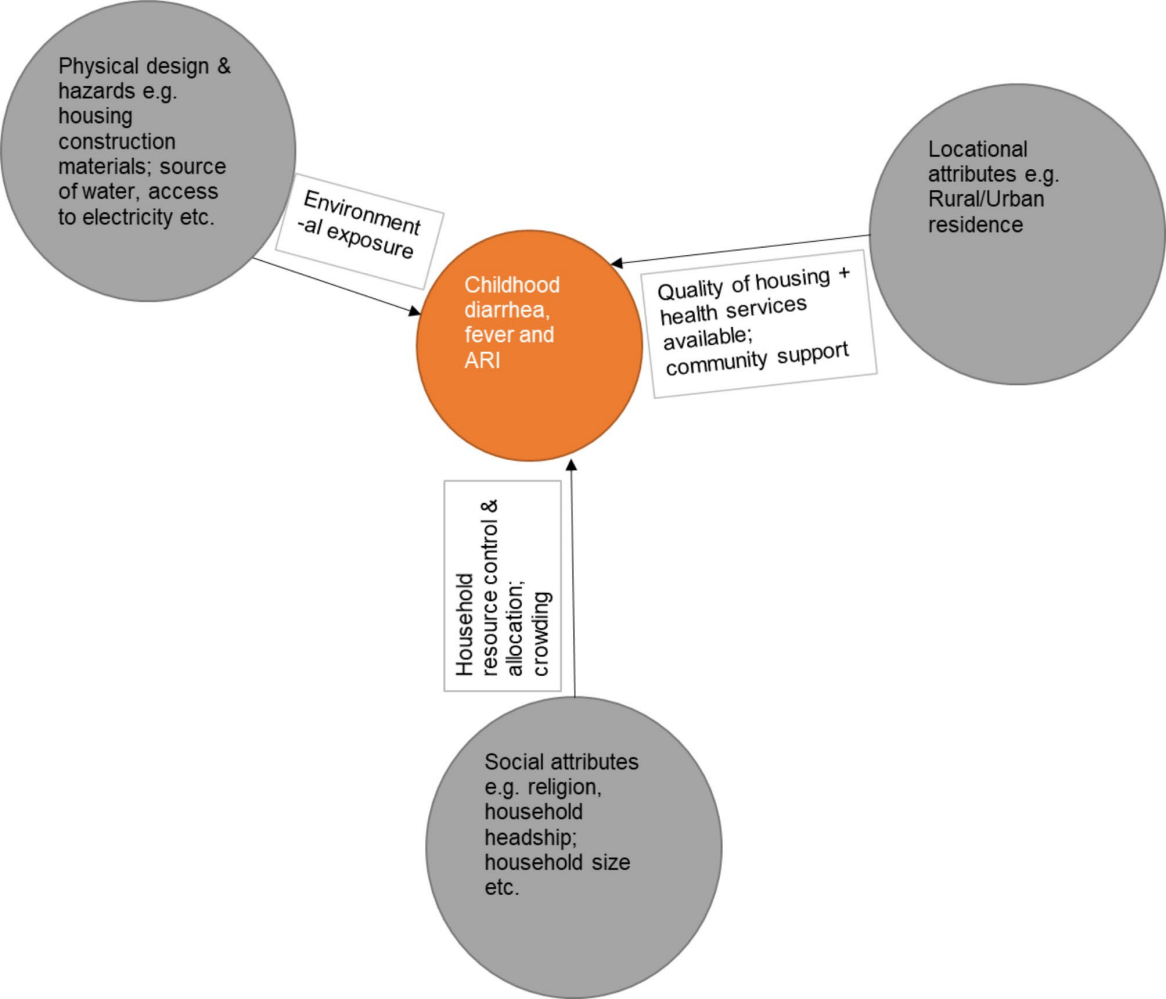
Conceptual framework on housing and child health outcomes. (adapted from Dunn 2020)

Based on available data, we revise the framework to include four attributes, except the financial and psychological attributes (see Fig. 1). The healthy housing index combines the first and second attributes, which address both the physical design and hazards that inhabitants face from such sources as poor access to WASH, and kitchen emissions from biomass among others. Social attributes in our paper include religion, household headship and education variables, which are proxies for social support. Lastly, our locational attributes are represented by the urban/rural divide, which is general but can give measures of community cohesion/support mostly noted in rural areas while urban areas have weaker cohesion. In addition, health service availability and quality differ across rural and urban areas in Africa, with urban areas better served compared to rural areas. Similarly, access to WASH and electricity is better in urban areas compared to rural areas.

## Methods

### Study design and participants

We use the Demographic and Health Survey (DHS) data for six countries - Burkina Faso (2010), Cameroon (2011), Ghana (2014), Kenya (2014), Nigeria (2018), and South Africa (2016), where the most recent survey collected health outcome data, i.e. diarrhoea, acute respiratory illness (ARI), and fever for children under 5 years were harmonized at the time of analysis. The DHS was designed to collect data to facilitate the understanding of the population and health situations of over 90 participating countries for more than 30 years [19]. All countries under the DHS program, through their Ministry of Health, and Central Statistical Agencies, are required to maintain high ethical standards protecting survey participants, securing informed consent, and ensuring the privacy and anonymity of study participant is maintained. The study protocols are vetted by countries’ institutional review boards (IRBs) to ensure that there is minimal risk of harm to participants. Each country utilizes similar data collection tools to facilitate comparability across countries. Data are collected by trained interviewers, on a wide range of demographic and health outcomes for household members and household-level information. Particularly for the household questionnaire, source of drinking/non-drinking water, type of sanitation facilities, materials used to construct the house, ownership of various consumer goods, and other household-level information are collected through self-reports by respondents. Further in many countries, DHS provides the key data that detail trends in child mortality and indicators of population, nutrition, morbidity, and health outcomes. The data used in this research were extracted from the IPUMS-DHS International database [20]. A total sample size of 91,096 is used in the analysis (i.e. 15, 044 for Burkina Faso, 11, 732 for Cameroon, 5, 884 for Ghana, 20, 964 for Kenya, 33, 924 for Nigeria, and 3,548 for South Africa.

### Measurements

The study focuses on three outcomes, namely, diarrhoea, acute respiratory illness (ARI), and fever among children each coded yes/no to indicate the presence or absence of disease. The main exposure variable is the healthy housing status (quality housing). Tusting et al. used a binary categorization of housing with improved housing linked to having improved WASH indicators, sufficient housing area, and finished building materials. An alternative characterization of housing quality is developed by Iddi et al. [2] using a validated continuous composite index score, referred to as the Health Housing Index (HHI), which was developed using a wide range of indicators including WASH (source of drinking water, type of toilet), building materials (roof, wall, floor), and house environmental conditions (cooking fuel, smoking). The index was developed from a 1-factor analysis model. The details of the development and validation of the index are published in the paper by Iddi and colleagues [2]. In the present paper, the index is categorized by placing the quality of housing into quantiles to allow evaluation of quality housing at different levels. We refer to the lowest quintile as the unhealthier household (lowest quality) and the highest quintile as the healthier household (highest quality). We include 13 explanatory variables which are known to influence child morbidity outcomes (demographics, household-, mother, and child-level factors) as control variables for the statistical analysis. Residency is coded to have two levels, rural and urban. The age of the head of the household is categorized into < 24 years, 25–34 years, 35–49 years, and above 49 years. Mothers’ educational level factors considered include; education which is grouped into no, primary, secondary and higher education, Mother’s BMI status categorized into normal, obese, and overweight, mother’s marital status (never married, married, other), age grouped into 15–24 years, 25–34 years, and 35–49 years, and religious status classified into Christian, Muslims, No religion, and traditional/other. Child-level indicators include the child’s gender, age (categorized into < 1, 1,2, 3, and 4 years), whether the child is from multiple or single births, and breastfeeding status of the child categorized into ever breastfed, never breastfed, and still breastfeeding.

### Statistical analysis

Descriptive analyses in the form of numbers and percentages and cross-tabulations with a chi-square test of associations are conducted. Measures are presented at the national and sub-national levels and further disaggregated according to a series of demographic characteristics such as urban and rural areas [19]. We employ a survey-weighted logistic regression model to assess the association between housing quality using a proxy healthy housing index,^2^ and the health outcomes (diarrhoea, acute respiratory illness, and fever). The analyses were conducted using the R **svyglm** function in the “**survey**” package [21] with cluster and household within-cluster used as clusters and sample strata specified as the stratifying variable for each country.

## Results

Descriptive analyses indicate that the prevalence of diarrhoea was lowest in South Africa (11%) and highest in Cameroon (22%) while acute respiratory illness breathing was lowest in Burkina Faso (10%) and highest in Cameroon (37%). Lastly, fever was lowest in Ghana (14%) and highest in Cameroon (27%) (see Table 1).

**Table 1:** Prevalence of diarrhoea, cough, and fever

	Diarrhoea		Acute respiratory illness		Fever	
	No	Yes	No	Yes	No	Yes
Burkina Faso (2010)	11,826 (85%)	2064 (15%)	12,475 (90%)	1425 (10%)	11,012 (79%)	2886 (21%)
Cameroon (2011)	8095 (78%)	2243 (22%)	6484 (63%)	3865 (37%)	7597 (73%)	2778 (27%)
Ghana (2014)	4728 (88%)	638 (12%)	4627 (86%)	752 (14%)	4637 (86%)	752 (14%)
Kenya (2014)	15,674 (85%)	2844 (15%)	11,735 (63%)	6815 (37%)	13,989 (75%)	4562 (25%)
Nigeria (2018)	26,823 (87%)	3950 (13%)	26,213 (85%)	4568 (15%)	23,308 (76%)	7466 (24%)
South Africa (2016)	2880 (89%)	356 (11%)	2424 (74%)	847 (26%)	2581 (79%)	679 (21%)

From Table 2, when prevalence is assessed by the healthy housing category, it is observed that most children with diarrhoea and fever resided in unhealthy homes with the prevalence of diarrhoea ranging from 13% in South Africa to 32% in Cameroon while fever ranged from 16% in South Africa to 31% in Nigeria and Cameroon. For acute respiratory illness, the prevalence was higher in healthy homes ranging from 10% in Burkina Faso to 46% in Cameroon. Kenya and Cameroon however had a high prevalence of ARI across all types of housing.

**Table 2:** Health outcomes and healthy housing status

	Diarrhoea					
	Unhealthier	Unhealthy	Healthy	Healthier	Healthiest	p- value
Burkina Faso (2010)	275 (14%)	526 (14%)	477 (15%)	419 (16%)	276 (17%)	0.0228
Cameroon (2011)	722 (32%)	511 (21%)	452 (21%)	265 (16%)	296 (15%)	0.0000
Ghana (2014)	134 (13%)	165 (16%)	127 (11%)	101 (11%)	88 (9%)	0.0000
Kenya (2014)	703 (18%)	682 (17%)	499 (15%)	452 (15%)	504 (13%)	0.0000
Nigeria (2018)	1301 (18%)	1051 (15%)	678 (12%)	552 (10%)	387 (7%)	0.0000
South Africa (2016)	96 (13%)	84 (14%)	65 (10%)	23 (8%)	80 (9%)	0.0001
	**Acute respiratory illness**					
Burkina Faso (2010)	175 (9%)	309 (8%)	309 (10%)	293 (11%)	279 (17%)	0.0000
Cameroon (2011)	813 (37%)	797 (32%)	749 (35%)	599 (36%)	912 (46%)	0.0000
Ghana (2014)	136 (13%)	129 (12%)	143 (13%)	145 (16%)	165 (16%)	0.0293
Kenya (2014)	1372 (35%)	1695 (41%)	1352 (39%)	1108 (36%)	1278(32%)	0.0000
Nigeria (2018)	1151 (16%)	996 (14%)	841 (15%)	768 (14%)	815 (15%)	0.0012
South Africa (2016)	149 (21%)	149 (25%)	196 (30%)	80 (30%)	251 (27%)	0.0008
	**Fever**					
Burkina Faso (2010)	387 (20%)	714 (19%)	688 (22%)	637 (24%)	363 (22%)	0.0000
Cameroon (2011)	701 (31%)	638 (26%)	578 (27%)	410 (25%)	454 (23%)	0.0000
Ghana (2014)	177 (17%)	162 (15%)	151 (13%)	127 (14%)	110 (11%)	0.0010
Kenya (2014)	1020 (26%)	1210 (29%)	869 (25%)	688 (22%)	773 (19%)	0.0000
Nigeria (2018)	2204 (31%)	2007 (28%)	1333 (23%)	1123 (21%)	814 (15%)	0.0000
South Africa (2016)	115 (16%)	121 (20%)	156 (24%)	65 (24%)	202 (21%)	0.0024

**Table 3a:** Association between child diarrhoea and risk factors (Adjusted odds ratios and confidence intervals)

Terms	Ghana (2014)	Burkina Faso (2010)	Cameroon (2011)	Kenya (2014)	Nigeria (2018)	South Africa (2016)
(Intercept)	0.13(0.03, 0.47)	0.19(0.07,0.53)	0.39(0.17,0.92)	0.31(0.18,0.55)	0.19(0.11,0.33)	0.22(0.02, 2.76)
**Healthy housing status(ref: Unhealthier)**						
Healthier	1.21(0.66, 2.20)	1.24(0.92,1.66)	**0.50(0.35,0.70)**	**0.79(0.63,0.98)**	**0.61(0.50,0.74)**	0.37(0.09, 1.54)
Healthiest	0.95(0.50, 1.81)	1.34(0.82,2.18)	**0.48(0.32,0.71)**	**0.68(0.52,0.87)**	**0.48(0.37,0.62)**	0.59(0.21, 1.61)
Healthy	0.96(0.58, 1.58)	1.21(0.90,1.64)	**0.60(0.44,0.83)**	**0.76(0.62,0.91)**	**0.71(0.59,0.86)**	**0.41(0.18, 0.97)**
Unhealthy	1.43(0.94, 2.17)	1.02(0.77,1.35)	**0.60(0.44,0.81)**	0.84(0.70,1.02)	**0.78(0.67,0.91)**	1.40(0.72, 2.71)
**Household head age (Ref: 0-24years)**						
25–34 years	1.27(0.65, 2.47)	0.89(0.59,1.33)	1.55(0.93,2.60)	0.93(0.73,1.18)	0.89(0.68,1.16)	0.86(0.27, 2.81)
35–49 years	1.22(0.60, 2.48)	0.71(0.47,1.08)	1.31(0.78,2.21)	0.85(0.67,1.08)	0.83(0.64,1.09)	0.54(0.13, 2.18)
50 + years	1.22(0.54, 2.74)	0.85(0.54,1.33)	1.69(0.98,2.90)	0.81(0.63,1.05)	0.83(0.62,1.12)	0.67(0.16, 2.71)
**Breastfeeding status (Ref: Ever breastfed)**						
Never breastfed	1.00(0.20, 5.13)	**2.10(1.09,4.05)**				
Still breastfeeding	1.18(0.72, 1.94)	1.22(0.93,1.61)				
**Mother’s Education level (Ref: No education)**						
Higher	0.32(0.09, 1.12)	1.04(0.19,5.59)	**0.38(0.20,0.75)**	**0.68(0.48,0.96)**	0.79(0.61,1.04)	0.79(0.19, 3.33)
Primary	0.89(0.58, 1.37)	1.23(0.96,1.58)	**0.67(0.54,0.83)**	**1.33(1.09,1.63)**	1.11(0.94,1.30)	0.54(0.13, 2.34)
Secondary	0.77(0.53, 1.11)	0.95(0.63,1.44)	**0.48(0.35,0.66)**	**1.28(1.01,1.62)**	1.03(0.87,1.20)	0.50(0.15, 1.66)
**Residence (Ref: Rural)**						
Urban	1.03(0.69, 1.54)	0.89(0.62,1.26)	1.18(0.90,1.56)	1.15(0.97,1.36)	0.91(0.77,1.08)	0.99(0.53, 1.86)
**Head of HH (Ref: Female)**						
Male	0.98(0.65, 1.48)	1.15(0.84,1.57)	1.15(0.88,1.51)	0.99(0.87,1.13)	1.06(0.88,1.28)	1.43(0.85, 2.40)
**Mother’s BMI (Ref: Underweight)**						
Normal	1.28(0.65, 2.49)	0.83(0.67,1.03)	**0.57(0.41,0.80)**	0.78(0.59,1.04)		1.25(0.26, 6.05)
Obese	3.46(0.56,21.40)	0.57(0.24,1.32)	**0.40(0.19,0.83)**	0.60(0.29,1.24)		1.64(0.26,10.35)
Overweight	1.04(0.29, 3.71)	0.70(0.34,1.42)	**0.43(0.27,0.69)**	0.71(0.48,1.06)		1.83(0.35, 9.48)
**Child age (Ref: below 1 year)**						
1 year	**2.40(1.60, 3.61)**	**1.76(1.42,2.18)**	**2.46(1.89,3.21**)	**1.26(1.08,1.46)**	**1.52(1.36,1.70)**	**1.36(0.69, 2.66)**
2 years	**2.59(1.43, 4.70)**	**1.55(1.14,2.12)**	1.31(0.94,1.81)	**0.76(0.65,0.88)**	0.97(0.85,1.09)	**0.47(0.23, 0.95)**
3 years	0.78(0.36, 1.72)	0.84(0.57,1.23)	0.85(0.64,1.13)	**0.41(0.34,0.49)**	**0.55(0.48,0.63)**	0.55(0.22, 1.38)
4 years	1.02(0.49, 2.12)	**0.44(0.29,0.67)**	**0.52(0.36,0.74)**	**0.29(0.24,0.36)**	**0.42(0.36,0.48)**	0.47(0.22, 1.00)
**Child sex (Ref: Male)**						
Female	**0.67(0.51, 0.89)**	0.95(0.82,1.11)	0.93(0.80,1.09)	**0.88(0.79,0.98)**	1.02(0.94,1.10)	0.74(0.45, 1.22)
**Multiple births(Ref: Yes)**						
No	0.71(0.35, 1.44)	1.27(0.71,2.25)	0.94(0.61,1.45)	1.03(0.70,1.53)	1.00(0.75,1.33)	1.74(0.46, 6.56)
**Mother’s marital status (Ref: never married)**						
Married	0.55(0.29, 1.04)	0.70(0.37,1.33)	1.53(0.98,2.37)	1.16(0.91,1.49)	0.81(0.54,1.24)	0.84(0.40, 1.74)
Others	0.67(0.36, 1.25)	1.21(0.59,2.47)	**1.75(1.12,2.73)**	**1.61(1.24,2.09)**	0.87(0.57,1.33)	1.61(0.86, 3.00)
**Mother’s age (Ref: 15–24 years)**						
25–34 years	1.06(0.76, 1.50)	0.94(0.76,1.16)	0.84(0.64,1.09)	0.90(0.77,1.05)	0.97(0.87,1.09)	1.25(0.67, 2.34)
35–49 years	1.54(0.72, 3.28)	0.92(0.54,1.55)	0.71(0.38,1.30)	0.98(0.67,1.44)	1.05(0.80,1.37)	0.77(0.08, 7.33)
**Mother’s religion (Ref: Christian)**						
Muslim	**1.90(1.36, 2.66**)	1.09(0.91,1.30)	1.16(0.93,1.44)	0.93(0.74,1.18)	2.16(1.89,2.48)	
No religion	0.84(0.38, 1.88)	0.19(0.03,1.09)	0.85(0.51,1.41)	**1.54(1.17,2.02)**		
Traditional/other	0.92(0.33, 2.55)	1.06(0.75,1.51)	0.91(0.61,1.37)	0.89(0.38,2.08)	0.46(0.19,1.11)	

**Table 3b:** Association between childhood acute respiratory illness and risk factors (Adjusted odds ratios and confidence intervals)

Terms	Ghana (2014)	Burkina Faso (2010)	Cameroon (2011)	Kenya (2014)	Nigeria (2018)	South Africa (2016)
(Intercept)	0.14(0.03,0.64)	0.09(0.02,0.37)	0.65(0.26,1.63)	0.25(0.14,0.44)	0.29(0.18,0.47)	0.02(0.00, 0.20)
**Healthy housing status(ref: Unhealthier)**						
Healthier	1.03(0.60,1.76)	1.40(0.96,2.05)	0.72(0.51,1.02)	**0.81(0.69,0.95)**	**0.72(0.60,0.87)**	1.91(0.76, 4.84)
Healthiest	1.07(0.61,1.88)	**2.45(1.39,4.34)**	0.98(0.67,1.43)	**0.66(0.54,0.81)**	**0.69(0.56,0.85)**	1.64(0.78, 3.49)
Healthy	0.71(0.44,1.16)	**1.55(1.09,2.20)**	**0.72(0.54,0.96)**	0.94(0.81,1.08)	**0.78(0.66,0.92)**	**2.36(1.31, 4.25)**
Unhealthy	0.74(0.45,1.20)	1.10(0.75,1.62)	0.86(0.67,1.12)	0.98(0.85,1.12)	**0.80(0.69,0.93)**	1.39(0.83, 2.33)
**Household head age (Ref: 0-24years)**						
25–34 years	1.05(0.55,2.00)	0.74(0.47,1.14)	1.12(0.57,2.23)	0.94(0.78,1.14)	0.95(0.74,1.23)	0.83(0.39, 1.74)
35–49 years	0.88(0.49,1.58)	0.76(0.47,1.22)	0.95(0.48,1.89)	0.92(0.76,1.12)	0.93(0.72,1.21)	1.03(0.45, 2.33)
50 + years	0.93(0.46,1.91)	0.61(0.37,1.01)	0.99(0.50,1.94)	0.98(0.79,1.21)	0.84(0.64,1.10)	0.93(0.39, 2.19)
**Breastfeeding status (Ref: Ever breastfed)**						
Never breastfed	2.44(0.67,8.89)	1.56(0.81,2.99)				
Still breastfeeding	1.35(0.78,2.32)	1.07(0.77,1.47)				
**Mother’s Education level (Ref: No education)**						
Higher	0.55(0.23,1.30)	0.35(0.04,2.69)	1.44(0.85,2.44)	**1.64(1.25,2.14)**	**1.38(1.14,1.68)**	2.98(0.87,10.22)
Primary	0.82(0.51,1.31)	0.96(0.72,1.29)	0.91(0.72,1.17)	**1.88(1.56,2.27)**	**1.39(1.20,1.62)**	3.68(1.12,12.11)
Secondary	0.82(0.52,1.29)	1.15(0.78,1.69)	1.02(0.77,1.36)	**2.06(1.67,2.54)**	**1.44(1.26,1.64)**	2.66(0.89, 7.97)
**Residence (Ref: Rural)**						
Urban	1.07(0.75,1.53)	1.21(0.78,1.89)	**1.40(1.09,1.80)**	1.08(0.95,1.23)	0.89(0.76,1.03)	0.87(0.49, 1.55)
**Head of HH (Ref: Female)**						
Male	0.89(0.65,1.22)	**1.67(1.06,2.61)**	1.18(0.96,1.45)	1.00(0.90,1.11)	1.09(0.93,1.27)	1.46(0.90, 2.38)
**Mother’s BMI (Ref: Underweight)**						
Normal	1.09(0.58,2.02)	0.98(0.77,1.25)	0.90(0.67,1.20)	**1.28(1.01,1.62)**		1.54(0.50, 4.75)
Obese	**0.12(0.01,0.97)**	1.18(0.50,2.78)	0.91(0.55,1.52)	0.87(0.51,1.47)		1.27(0.31, 5.13)
Overweight	0.99(0.32,3.10)	0.47(0.22,1.00)	0.92(0.63,1.36)	1.16(0.85,1.58)		1.42(0.43, 4.68)
**Child age (Ref: below 1 year)**						
1 year	**1.64(1.04,2.61)**	1.17(0.90,1.52)	**1.23(1.00,1.52)**	**1.35(1.19,1.54)**	**1.25(1.13,1.39)**	**1.70(1.00, 2.91)**
2 years	1.78(0.96,3.29)	0.95(0.67,1.37)	0.92(0.74,1.15)	**1.21(1.08,1.37)**	0.89(0.80,1.00)	**1.68(1.01, 2.79)**
3 years	1.46(0.72,2.97)	0.73(0.48,1.10)	0.99(0.81,1.22)	**1.15(1.02,1.30)**	**0.84(0.75,0.93)**	1.16(0.65, 2.06)
4 years	1.14(0.54,2.41)	**0.45(0.28,0.70)**	0.88(0.69,1.11)	1.06(0.92,1.22)	**0.63(0.56,0.71)**	0.93(0.58, 1.49)
**Child sex (Ref: Male)**						
Female	0.98(0.75,1.27)	0.91(0.77,1.08)	0.97(0.85,1.11)	1.06(0.98,1.15)	1.01(0.94,1.08)	1.19(0.84, 1.68)
**Multiple births(Ref: Yes)**						
No	1.60(0.63,4.10)	0.72(0.40,1.31)	0.88(0.59,1.33)	0.96(0.68,1.36)	0.94(0.74,1.21)	1.71(0.53, 5.57)
**Mother’s marital status (Ref: never married)**						
Married	0.66(0.37,1.18)	1.12(0.43,2.95)	1.08(0.77,1.52)	1.16(0.95,1.41)	0.83(0.62,1.11)	0.64(0.37, 1.10)
Others	0.72(0.39,1.31)	1.83(0.67,4.98)	**1.63(1.13,2.37)**	1.13(0.91,1.39)	0.91(0.65,1.28)	1.62(0.96, 2.73)
**Mother’s age (Ref: 15–24 years)**						
25–34 years	0.81(0.56,1.18)	**1.37(1.11,1.69)**	0.94(0.77,1.15)	1.00(0.89,1.12)	0.99(0.89,1.10)	0.95(0.60, 1.52)
35–49 years	0.89(0.40,1.99)	0.78(0.40,1.51)	1.11(0.67,1.84)	0.83(0.63,1.11)	1.07(0.85,1.34)	1.07(0.27, 4.18)
**Mother’s religion (Ref: Christian)**						
Muslim	1.08(0.75,1.55)	1.01(0.80,1.26)	1.11(0.87,1.41)	**0.66(0.54,0.81)**	**0.82(0.72,0.94)**	
No religion	0.70(0.30,1.63)	1.79(0.47,6.86)	0.75(0.45,1.23)	1.08(0.84,1.39)		
Traditional/other	**0.32(0.13,0.80)**	**1.52(1.06,2.18**)	0.76(0.53,1.10)	1.11(0.57,2.15)	0.42(0.17,1.01)	

**Table 3c:** Association between child fever and risk factors (Adjusted odds ratios and confidence intervals)

Terms	Ghana (2014)	Burkina Faso (2010)	Cameroon (2011)	Kenya (2014)	Nigeria (2018)	South Africa (2016)
(Intercept)	0.16(0.04,0.69)	0.55(0.24,1.25)	0.41(0.18,0.93)	0.34(0.20,0.58)	0.29(0.19,0.44)	0.01(0.00, 0.10)
**Healthy housing status(ref: Unhealthier)**						
Healthier	0.66(0.39,1.10)	1.28(0.97,1.70)	**0.67(0.49,0.92)**	**0.71(0.58,0.88)**	**0.70(0.59,0.82)**	1.83(0.71, 4.74)
Healthiest	**0.60(0.36,0.99**)	1.31(0.84,2.04)	**0.47(0.32,0.69)**	**0.64(0.49,0.82)**	**0.52(0.42,0.64)**	**2.09(1.02, 4.29)**
Healthy	0.86(0.54,1.36)	1.22(0.94,1.59)	**0.71(0.53,0.94)**	**0.82(0.69,0.98)**	**0.78(0.67,0.90)**	1.73(0.94, 3.18)
Unhealthy	0.96(0.66,1.39)	0.91(0.71,1.16)	**0.76(0.58,0.99)**	0.99(0.82,1.18)	0.90(0.79,1.01)	1.11(0.64, 1.94)
**Household head age (Ref: 0-24years)**						
25–34 years	0.60(0.29,1.23)	0.82(0.59,1.14)	1.02(0.58,1.80)	0.84(0.69,1.01)	0.82(0.66,1.01)	0.56(0.18, 1.73)
35–49 years	0.68(0.33,1.39)	0.72(0.52,1.02)	0.91(0.51,1.63)	0.82(0.67,1.01)	0.80(0.65,1.00)	1.16(0.42, 3.25)
50 + years	0.51(0.24,1.11)	0.80(0.55,1.14)	1.05(0.60,1.84)	0.94(0.75,1.17)	0.78(0.62,0.97)	0.98(0.37, 2.60)
**Breastfeeding status (Ref: Ever breastfed)**						
Never breastfed	0.64(0.14,2.83)	1.34(0.73,2.45)				
Still breastfeeding	1.55(0.94,2.57)	1.07(0.84,1.36)				
**Mother’s Education level (Ref: No education)**						
Higher	0.52(0.18,1.49)	**0.14(0.03,0.66)**	1.23(0.72,2.12)	**1.49(1.11,2.00)**	**0.76(0.62,0.92)**	1.00(0.29, 3.41)
Primary	1.05(0.70,1.56)	1.14(0.92,1.41)	0.89(0.70,1.13)	**1.78(1.44,2.20)**	1.04(0.92,1.17)	0.85(0.25, 2.91)
Secondary	0.79(0.52,1.21)	0.99(0.68,1.45)	0.96(0.71,1.28)	**1.71(1.35,2.16)**	1.03(0.91,1.15)	1.05(0.35, 3.11)
**Residence (Ref: Rural)**						
Urban	1.05(0.70,1.56)	0.94(0.72,1.24)	1.19(0.90,1.59)	1.03(0.88,1.20)	**0.82(0.71,0.94)**	0.84(0.47, 1.51)
**Head of HH (Ref: Female)**						
Male	0.74(0.52,1.06)	1.25(0.93,1.68)	1.24(0.98,1.57)	1.01(0.90,1.13)	1.12(0.98,1.29)	1.49(0.92, 2.40)
**Mother’s BMI (Ref: Underweight)**						
Normal	1.00(0.52,1.91)	0.94(0.76,1.16)	**0.72(0.52,0.99)**	0.84(0.66,1.07)		0.91(0.29, 2.84)
Obese	0.30(0.07,1.28)	0.94(0.49,1.77)	0.66(0.38,1.13)	0.60(0.33,1.08)		1.17(0.29, 4.82)
Overweight	0.36(0.10,1.32)	**0.55(0.34,0.91)**	**0.46(0.30,0.70)**	**0.61(0.44,0.85)**		0.90(0.26, 3.13)
**Child age (Ref: below 1 year)**						
1 year	**2.78(1.79,4.31)**	**1.90(1.56,2.32)**	**1.40(1.14,1.72)**	**1.36(1.18,1.55)**	**1.55(1.40,1.71)**	1.70(0.96, 3.01)
2 years	**3.87(2.16,6.95)**	**1.37(1.06,1.78)**	**1.25(1.02,1.54)**	1.04(0.91,1.18)	**1.31(1.19,1.44)**	**1.99(1.14, 3.48)**
3 years	**2.95(1.49,5.82)**	0.81(0.60,1.09)	1.20(0.98,1.48)	**0.86(0.75,0.99)**	1.08(0.98,1.19)	1.36(0.73, 2.55)
4 years	**3.10(1.60,5.99)**	0.63(0.45,0.88)	0.90(0.69,1.18)	**0.84(0.73,0.97)**	0.91(0.82,1.00)	0.62(0.31, 1.25)
**Child sex (Ref: Male)**						
Female	0.80(0.60,1.05)	0.96(0.84,1.10)	0.82(0.71,0.95)	0.99(0.91,1.08)	**1.06(1.00,1.13)**	0.92(0.60, 1.41)
**Multiple births(Ref: Yes)**						
No	1.18(0.51,2.75)	0.91(0.57,1.45)	1.13(0.77,1.68)	0.98(0.70,1.37)	1.11(0.90,1.37)	**25.84(3.26,204.67)**
**Mother’s marital status (Ref: never married)**						
Married	0.76(0.41,1.41)	**0.47(0.26,0.82)**	1.36(0.92,2.01)	1.04(0.83,1.29)	1.02(0.78,1.34)	0.77(0.45, 1.34)
Others	0.69(0.36,1.34)	0.60(0.32,1.13)	**1.60(1.07,2.40)**	0.99(0.78,1.26)	1.08(0.79,1.47)	1.28(0.70, 2.34)
**Mother’s age (Ref: 15–24 years)**						
25–34 years	1.05(0.73,1.50)	**1.30(1.09,1.56)**	1.01(0.81,1.27)	1.04(0.91,1.19)	**1.09(1.00,1.19)**	0.94(0.58, 1.53)
35–49 years	1.83(0.97,3.46)	1.40(0.93,2.12)	1.60(0.86,2.97)	1.12(0.84,1.51)	0.97(0.79,1.21)	**0.13(0.02, 0.96)**
**Mother’s religion (Ref: Christian)**						
Muslim	1.24(0.83,1.86)	0.92(0.78,1.09)	**1.24(1.00,1.53**)	0.77(0.61,0.98)	**1.37(1.20,1.56)**	
No religion	1.01(0.56,1.84)	**0.40(0.17,0.93**)	0.88(0.53,1.47)	1.21(0.89,1.64)		
Traditional/other	1.00(0.53,1.85)	1.11(0.86,1.44)	0.67(0.42,1.06)	1.22(0.44,3.39)	0.75(0.43,1.28)	

## Assessing the association between healthy housing and child health outcomes

We present the regression output separately for each of the three health outcomes. We conduct both unadjusted and adjusted analyses of the association between healthy housing status and health outcomes. In Table 3a and 3b, and in Appendix A1 and A3, both adjusted and unadjusted results generally indicate low odds of diarrhoea and fever among children living in healthy homes as compared to those living in unhealthier homes. For acute respiratory illness, unadjusted estimates, shown in Appendix A2, indicate elevated odds while after adjustment, children living in healthy homes were less likely to report having an episode of ARI.

Secondly, we focus on the analyses that include other determinants of the three health outcomes. For diarrhoea, healthy housing status, child’s breastfeeding status, age and sex; mother’s education, BMI, marital status, and religion were statistically significant. Healthy housing status was associated with reduced odds of diarrhoea in Cameroon, Kenya, South Africa, and Nigeria. In Burkina Faso, children who were never breastfed were almost two times more likely to have diarrhoea compared with those who were breastfed but had ceased breastfeeding at the time of the survey. Children born to mothers with any education had lower odds of having diarrhoea in Cameroon compared with children born to mothers without education. In Kenya, higher maternal education (post-secondary) was protective against diarrhoea, while primary and secondary levels of education were associated with increased odds of diarrhoea. Maternal BMI was only statistically significant in Cameroon where it was associated with lower odds of diarrhoea for normal, overweight, and obese categories compared to those in the underweight category.

Further, child age, specifically those aged one year were at increased odds of getting diarrhoea across all countries. On the other hand, two-year-old children had increased odds of diarrhoea in Ghana and Burkina Faso, and reduced odds in Kenya and South Africa. Further, three-year-old children in Kenya and Nigeria; and four-year-olds in all countries except Ghana and South Africa where this was not significant, had reduced odds of diarrhoea. Female children in Ghana and Kenya had reduced odds of diarrhoea compared with male children, while marital status (others compared to those never married) was associated with elevated odds of diarrhoea in Cameroon and Kenya. Lastly, children born to mothers professing the Islamic faith and those with no religious affiliation had increased odds of diarrhoea in Ghana and Kenya respectively, compared with those professing the Christian faith. These results are presented in Table 3a.

For acute respiratory illness, the mother’s education level, age, marital status, religion, BMI, healthy housing status, sex of household head, place of residence, and child age, were significantly associated with the outcome. In Kenya and Nigeria, children born to mothers with primary, secondary and higher education had increased odds of acute respiratory illness, with a secondary level of education having the highest odds of ARI. Mothers aged 25–34 years in Burkina Faso had children with increased odds of ARI compared with those aged 15–24 years while in the rest of the countries, this did not achieve statistical significance. In Cameroon, children of mothers in other marital arrangements versus never married mothers had increased odds of ARI. Further, religion was associated with reduced odds in Kenya and Nigeria for those professing the Islamic faith and in Ghana among those traditional/other faiths, while those with traditional/other religious affiliations in Burkina Faso had increased odds of acute respiratory illness. In Ghana, mothers who were obese had children with lower odds of ARI while in Kenya, normal maternal BMI was associated with increased odds of ARI. Healthy housing was associated with reduced odds in Cameroon, Kenya, and Nigeria while it was associated with increased odds in Burkina Faso and South Africa. Urban residence was associated with increased odds of acute respiratory illness in Cameroon, while children living in male-headed households in Burkina Faso had increased odds of ARI. Finally, children aged one year had increased odds of acute respiratory illness in all countries except Burkina Faso, compared with children aged below one year. Older children (three years and older) had lower odds of ARI in Burkina Faso and Nigeria. In Kenya, all ages were associated with increased odds of ARI (See Table 3b).

Lastly, healthy housing status, place of residence, mother’s education, age, BMI, marital status, education, religion, child age, and sex were significantly associated with fever (see Table 3c). Healthy housing was significantly associated with reduced odds of fever among children in all countries except South Africa where children living in the healthiest homes had more than double the odds of having fever. Urban residence in Nigeria was associated with lower odds of fever, while in the rest of the countries, it did not achieve statistical significance. In Kenya, maternal education (primary, secondary, and higher) was associated with increased odds of fever among children, while higher education was associated with reduced odds in Nigeria and Burkina Faso. Younger mothers (25–34 years) in Burkina Faso and Nigeria were more likely to have children who had a fever while the oldest age group (35–49 years) had lower odds in South Africa. Children born to mothers in other marital arrangements in Cameroon had increased odds of fever, while those born to married mothers had lower odds of fever in Burkina Faso. Children born to mothers with normal BMI had reduced odds of fever in Cameroon as were those born to overweight mothers in Burkina Faso, Cameroon, and Kenya. Children born to mothers professing the Islamic faith had increased odds of fever in Cameroon and Nigeria while those born to mothers without a religious affiliation had reduced odds of fever in Burkina Faso. Further, child age was associated with increased odds of fever for all ages (1–4 years) in Ghana, and for ages 1 and 2 years in Burkina Faso, Cameroon and Nigeria. In Kenya, only children aged 1 year had increased odds of fever, while those aged three and four years had reduced odds. Lastly, in South Africa, only two-year-olds had significantly increased odds of fever. Girls had increased odds of fever in Nigeria.

## Discussion

Our study assessed the role of housing on health outcomes among children aged below five years, and the findings indicate that housing is an important determinant of the three outcomes investigated, namely: diarrhoea, acute respiratory illness, and fever. Other factors associated with these health outcomes include community, household, child, and maternal level factors. Household-level factors besides healthy housing include the age of household head, and the community-level factor associated with child morbidity outcomes is the place of residence. Child-level factors identified include breastfeeding status, age, and sex. Lastly, maternal-level factors associated with morbidity outcomes include education, age, BMI, marital status, and religion.

Healthy housing was generally associated with reduced odds of ill health for all three outcomes considered, though this was not significant in all countries. The healthy housing status considered several attributes such as sanitation, drinking water sources as well as housing characteristics among others. Therefore, for housing that was considered healthy, there is noted net reduced odds of being ill for under-five children. This is attributed to better-constructed homes that protect occupants from the elements, ensure access to adequate space, and reduced overcrowding, as well as the use of cleaner cooking and lighting fuels, leading to reductions in household air pollution and therefore lower incidence of respiratory infections [22, 23]. Further, access to adequate sanitation and hygiene services and clean water sources have been known to reduce the incidence of diarrheal diseases [24, 25]. Tusting et al. [15], found reductions in diseases that are leading causes of child mortality including diarrhoea and respiratory illness among children living in housing with some of the attributes we considered in the healthy housing status i.e. improved drinking water, sanitation, and adequate space.

Child-level characteristics associated with the health outcomes include child age and breastfeeding status as well as sex. Children in Burkina Faso, who never breastfed were at heightened risk of diarrhoea compared to those who had stopped breastfeeding, in line with existing evidence on the protective role of breastfeeding over gastrointestinal and respiratory tract infections among children [26–29]. Increasing age of children appears to have been protective against diarrhoea, with one-year-olds having higher odds while four-year-olds were less likely to have diarrhoea. A multi-country study that assessed pathogen-specific diarrhoea in children found decreasing risk as children grew older, attributing this to immunity induced by previous infections [30]. The noted high risk among one-year-old children could also be driven by the introduction of solid foods, which coincides with reduced breastfeeding. Studies have indicated an increased risk of diarrhoea among children in LMICs due to the unhygienic conditions in which the food is prepared and stored [31]. Older children are likely to have a more developed immune system that confers protection against diarrhoea. Additionally, at one year, the children begin to explore their surroundings and the probability of ingesting infected materials may cause diarrhoea [32]. For ARI, though not significant in all countries, the results indicate a protective effect of age. It is likely that the younger children stay close to their caregivers who are in most cases female members of the household. In most African communities, younger children are strapped to the back of their caregivers even during episodes of cooking. With the use of biomass fuels that emit high levels of pollutants shown to have an effect on respiratory health, the presence of children in kitchens increases their exposure to these pollutants and therefore their risk of respiratory illness [29, 33–35]. For fever, children’s age was associated with increased risk in Ghana while in other countries where results were significant, the risk was reduced with increasing age. Increasing infections as children grow older and possibly begin attending school or day-care likely drive the observed patterns in Ghana. In Uganda, they found fever a co-morbidity of diarrhoea and ARI. The majority of fevers among children under-five are due to diarrhoea, followed by ARI. This calls for addressing causes of diarrhoea and ARI as pathways to addressing the prevalence of fever among under-5s. Furthermore, malaria, diarrhoea, and pneumonia have been identified as the top causes of under-five deaths in Uganda. These diseases present with fever as an indication of illness in the early stages. Furthermore, childhood fever is the most common clinical sign of infectious diseases. It is used as a measure of the disease public health burden, and of the effectiveness of programs aimed at preventing and treating diseases [36]. Lastly, female children were less likely to have diarrhoea in Kenya and more likely to have a fever in Nigeria, which confirms the sex differences in immune response reported in other studies [37, 38], as well as observed differences in the susceptibility and severity of infectious diseases in children [39].

Maternal age, education, marital status, and religion were associated with the health outcomes considered, with variations across countries. Children born to mothers in other marital arrangements (divorced, separated, widowed) had higher odds of diarrhoea in Cameroon and Kenya. Evidence from across Africa suggests that children’s health is influenced by their mother’s union status. Children of divorced mothers face lower odds of survival [40] and experience significant health disadvantages relative to their peers with married parents [41]. Mothers who were cohabiting were less likely to seek healthcare for childhood illnesses compared to married mothers [42] and married mothers are able to invest in health-seeking behaviors for their children more than unmarried mothers [43].

Lower maternal age has been associated with negative child outcomes attributed to inexperience in child rearing, especially among first-time mothers. As maternal age increases alongside the number of births, they gain critical experience in practices that protect their children from ill health [44]. While in some contexts any education level appears protective for diarrhoea, in others, only higher levels of education are protective. The mixed results for maternal education across countries may reflect country-specific education content as well as the economic returns to women’s education, affording highly educated women the ability to better look after their children. For acute respiratory illness, the higher risk of disease prevalence across education levels is counter-intuitive. Some of the risk factors for respiratory illnesses such as the use of highly polluting fuels like wood, charcoal, or kerosene may transcend educational levels due to cultural or individual preferences [45, 46]. Further, decision-making to transition to more expensive, low-emission fuels like liquefied petroleum gas (LPG) or biogas may fall on the (mostly male) head of household who may not have the financial ability to make this transition, or who may not see the need for change [47, 48]. Therefore, the education level of the woman would have little effect on these risk factors, leading to observed elevated odds of acute respiratory illness. In Kenya where education has no significant positive influence on fever morbidity, though counter-intuitive, is consistent with findings in the last decade that have raised questions on the functionality of education received, especially at lower levels. It points to how the limited availability of formal sector jobs and high rates of unemployment in the country has weakened the independent role of education in helping individuals move out of poverty [49]. This challenge is especially noted among informal workers and those living in informal settlements that have consistently shown that educational attainment might not independently pull households out of poverty [50]. Lastly for fever, existing evidence on the importance of a mother’s education on child health outcomes points to its protective effect [51, 52]. However, it appears that in some contexts, having primary education is as risky to child health outcomes as having no education at all, possibly an indication of the quality of education at the primary level. Education offers critical information on child health including how to identify the symptoms and what actions mothers can take to prevent disease. Further, education provides an opportunity for employment, giving mothers the financial ability to take proper care of their children.

Finally, religion has a strong influence on health and our findings indicate mixed results on the association with diarrhoea, ARI, and fever. Religion has a bearing on women’s control over household affairs and decision-making [53]. Some religious groups may encourage polygamy, which has been shown to have a negative effect on child health, especially the incidence of diarrhoea and respiratory illness due to the higher odds of having other under-5 children in the home, a factor that increases the transmission of these conditions [54]. In addition, mothers in polygamous marriages have also been shown to have lower educational attainment, which negatively affects child health [54], as well as lower bargaining power to influence resource allocation [55], which may impact the overall health of their children. Further, religion has been found to increase the uptake of childhood immunization, which influences the incidence of childhood diarrhoea [56].

## Conclusion and recommendations

In conclusion, we have demonstrated the utility of a new approach to quantifying quality housing and assessed its association with child health outcomes. The use of data from several countries in SSA provides a regional overview of the effect of housing quality on child health outcomes. The heterogeneity of findings across similar covariates and the multiple relations between healthy housing and under-5 morbidity patterns show unequivocally the heterogeneity that exists across African countries and the need to account for different contexts in efforts to seek an understanding of the role of healthy housing in child morbidity and general health outcomes. While healthy housing is significantly associated with positive morbidity outcomes across the six African countries, our findings beyond the commonalities identified, have also shown considerable variations *within* and across countries in the region that is associated with the net role of broader social determinants of health-related to environmental factors, socioeconomic status, and individual levels characteristics. This calls for bold but nuanced policy and program interventions in understanding and addressing beyond healthy housing, the challenge of morbidity and health outcomes among under-five children in the SSA region. Reflecting on the conceptual framework, we recommend that national health and demographic surveys should include sufficient data that captures the six attributes proposed in Dunn’s framework.

## Study limitations

Comparison of results in all the countries involved in this analysis was limited due to different years during data collection. Additionally, our analysis did not include data on financial and psychological attributes from Dunn’s framework due to the unavailability of such data.

## Electronic supplementary material

Below is the link to the electronic supplementary material.

Supplementary Material 1

## Data Availability

Anonymized data were obtained from the IPUMS International database (Minnesota Population Center (MPC, 2020) [20].
