# Supplementary material for: Housing and health outcomes: evidence on child morbidities from six Sub-Saharan African countries
Source: BMC Pediatr. 2023 May 5;23:219. doi: 10.1186/s12887-023-03992-5 (PMC10163804; doi:10.1186/s12887-023-03992-5)
Supplement: Supplementary file 1 — Supplementary Material 1 [file 12887_2023_3992_MOESM1_ESM.docx]

Housing and Health Outcomes: Evidence on Child Morbidities from Six Sub-Saharan African Countries.

Kanyiva Muindi^1*^, Samuel Iddi^1,2^*, Hellen Gitau^1^, Blessing Mberu^1,3^

^1^ Population Dynamics and Urbanization, African Population and Health Research Center, Nairobi, Kenya

^2^Department of Statistics and Actuarial Science, University of Ghana, Legon, Accra, Ghana

^3^Demography and Population Studies, University of Witwatersrand, Johannesburg South Africa.

*Correspondence: [siddi@ug.edu.gh](mailto:siddi@ug.edu.gh); [kmuindi@aphrc.org](mailto:kmuindi@aphrc.org)

**SUPPLEMENTARY MATERIAL**

Table A1: Association between child diarrhoea and healthy housing status

| Effects | **Unadjusted estimates** | | | | | |
| --- | --- | --- | --- | --- | --- | --- |
|  | Ghana (2014) | **Burkina Faso (2010)** | **Cameroon (2011)** | **Kenya (2014)** | **Nigeria (2018)** | **South Africa (2016)** |
| Intercept | 0.15(0.11,0.20) | 0.16(0.14,0.19) | 0.48(0.41,0.55) | 0.22(0.19,0.25) | 0.23(0.20,0.26) | 0.16(0.12,0.20) |
| **HH Quintile (ref: Unhealthier)** | | | | | | |
| Unhealthy | 1.25(0.88,1.79) | 0.97(0.81,1.18) | **0.55(0.45,0.68)** | 0.91(0.77,1.07) | **0.77(0.66,0.89)** | 1.07(0.73,1.56) |
| Healthy | 0.86(0.59,1.27) | 1.10(0.90,1.34) | **0.56(0.46,0.70)** | **0.78(0.65,0.92)** | **0.60(0.51,0.71)** | 0.72(0.50,1.04) |
| Healthier | 0.82(0.54,1.24) | 1.13(0.93,1.38) | **0.40(0.32,0.50)** | **0.78(0.64,0.94)** | **0.50(0.42,0.60)** | 0.60(0.31,1.17) |
| Healthiest | 0.64(0.39,1.04) | 1.24(0.97,1.59) | **0.37(0.29,0.47)** | **0.66(0.54,0.81)** | **0.33(0.26,0.41)** | **0.60(0.40,0.92)** |
|  | **Adjusted estimates** | | | | | |
| Intercept | 0.13(0.03, 0.47) | 0.19(0.07,0.53) | 0.39(0.17,0.92) | 0.31(0.18,0.55) | 0.19(0.11,0.33) | 0.22(0.02, 2.76) |
| **HH Quintile (ref: Unhealthier)** | |  | | | | |
| Unhealthy | 1.43(0.94, 2.17) | 1.02(0.77,1.35) | **0.60(0.44,0.81)** | 0.84(0.70,1.02) | **0.78(0.67,0.91)** | 1.40(0.72, 2.71) |
| Healthy | 0.96(0.58, 1.58) | 1.21(0.90,1.64) | **0.60(0.44,0.83)** | **0.76(0.62,0.91)** | **0.71(0.59,0.86)** | **0.41(0.18, 0.97)** |
| Healthier | 1.21(0.66, 2.20) | 1.24(0.92,1.66) | **0.50(0.35,0.70)** | **0.79(0.63,0.98)** | **0.61(0.50,0.74)** | 0.37(0.09, 1.54) |
| Healthiest | 0.95(0.50, 1.81) | 1.34(0.82,2.18) | **0.48(0.32,0.71)** | **0.68(0.52,0.87)** | **0.48(0.37,0.62)** | 0.59(0.21, 1.61) |

Table A2: Association between acute respiratory illness and healthy housing status

| Effects | **Unadjusted estimates** | | | | | |
| --- | --- | --- | --- | --- | --- | --- |
|  | Ghana (2014) | **Burkina Faso (2010)** | **Cameroon (2011)** | **Kenya (2014)** | **Nigeria (2018)** | **South Africa (2016)** |
| Intercept | 0.15(0.12,0.19) | 0.10(0.08,0.12) | 0.58(0.50,0.67) | 0.54(0.48,0.61) | 0.19(0.17,0.22) | 0.26(0.20,0.33) |
| **HH Quintile (ref: Unhealthier)** | | | | | | |
| Unhealthy | 0.93(0.69,1.26) | 0.89(0.68,1.15) | **0.83(0.70,0.99)** | **1.29(1.12,1.48)** | **0.83(0.72,0.97)** | 1.30(0.94,1.79) |
| Healthy | 0.97(0.70,1.35) | 1.11(0.87,1.41) | 0.94(0.77,1.13) | **1.19(1.03,1.38)** | 0.89(0.76,1.05) | **1.66(1.17,2.37)** |
| Healthier | 1.24(0.89,1.71) | 1.25(0.97,1.61) | 0.99(0.79,1.22) | 1.03(0.87,1.21) | 0.85(0.72,1.01) | **1.62(1.04,2.51)** |
| Healthiest | 1.28(0.89,1.84) | 2.08(1.56,2.76) | **1.47(1.20,1.81)** | 0.87(0.72,1.06) | 0.87(0.72,1.06) | 1.40(0.99,1.98) |
|  | **Adjusted estimates** | | | | | |
| Intercept | 0.13(0.03, 0.47) | 0.19(0.07,0.53) | 0.39(0.17,0.92) | 0.31(0.18,0.55) | 0.19(0.11,0.33) | 0.22(0.02, 2.76) |
| Unhealthy | 1.43(0.94, 2.17) | 1.02(0.77,1.35) | **0.60(0.44,0.81)** | 0.84(0.70,1.02) | **0.78(0.67,0.91)** | 1.40(0.72, 2.71) |
| Healthy | 0.96(0.58, 1.58) | 1.21(0.90,1.64) | **0.60(0.44,0.83)** | **0.76(0.62,0.91)** | **0.71(0.59,0.86)** | **0.41(0.18, 0.97)** |
| Healthier | 1.21(0.66, 2.20) | 1.24(0.92,1.66) | **0.50(0.35,0.70)** | **0.79(0.63,0.98)** | **0.61(0.50,0.74)** | 0.37(0.09, 1.54) |
| Healthiest | 0.95(0.50, 1.81) | 1.34(0.82,2.18) | **0.48(0.32,0.71)** | **0.68(0.52,0.87)** | **0.48(0.37,0.62)** | 0.59(0.21, 1.61) |

Table A3: Association between fever and healthy housing status

| Effects | **Unadjusted estimates** | | | | | |
| --- | --- | --- | --- | --- | --- | --- |
|  | Ghana (2014) | **Burkina Faso (2010)** | **Cameroon (2011)** | **Kenya (2014)** | **Nigeria (2018)** | **South Africa (2016)** |
| Intercept | 0.20(0.16,0.25) | 0.25(0.21,0.29) | 0.46(0.38,0.55) | 0.36(0.31,0.40) | 0.45(0.41,0.50) | 0.19(0.15,0.24) |
| **HH Quintile (ref: Unhealthier)** | | | | | | |
| Unhealthy | 0.88(0.65,1.19) | 0.93(0.78,1.11) | **0.77(0.63,0.94)** | 1.17(0.99,1.39) | **0.86(0.76,0.98)** | 1.36(0.96,1.92) |
| Healthy | 0.76(0.56,1.05) | 1.14(0.95,1.38) | 0.82(0.64,1.04) | 0.95(0.81,1.12) | **0.67(0.59,0.77)** | **1.67(1.18,2.37)** |
| Healthier | 0.77(0.53,1.12) | **1.27(1.05,1.53)** | **0.72(0.57,0.92)** | **0.81(0.66,0.98)** | **0.58(0.50,0.67)** | **1.68(1.09,2.57)** |
| Healthiest | **0.59(0.41,0.84)** | 1.16(0.92,1.46) | **0.65(0.51,0.82)** | **0.68(0.56,0.83)** | **0.37(0.31,0.45)** | **1.43(1.02,2.03)** |
|  | **Adjusted estimates** | | | | | |
| Intercept | 0.16(0.04,0.69) | 0.55(0.24,1.25) | 0.41(0.18,0.93) | 0.34(0.20,0.58) | 0.29(0.19,0.44) | 0.01(0.00, 0.10) |
| Unhealthy | 0.96(0.66,1.39) | 0.91(0.71,1.16) | **0.76(0.58,0.99)** | 0.99(0.82,1.18) | 0.90(0.79,1.01) | 1.11(0.64, 1.94) |
| Healthy | 0.86(0.54,1.36) | 1.22(0.94,1.59) | **0.71(0.53,0.94)** | **0.82(0.69,0.98)** | **0.78(0.67,0.90)** | 1.73(0.94, 3.18) |
| Healthier | 0.66(0.39,1.10) | 1.28(0.97,1.70) | **0.67(0.49,0.92)** | **0.71(0.58,0.88)** | **0.70(0.59,0.82)** | 1.83(0.71, 4.74) |
| Healthiest | **0.60(0.36,0.99)** | 1.31(0.84,2.04) | **0.47(0.32,0.69)** | **0.64(0.49,0.82)** | **0.52(0.42,0.64)** | **2.09(1.02, 4.29)** |
